# Commentary: Challenges and Priorities for Pediatric Critical Care Clinician–Researchers in Low- and Middle-Income Countries

**DOI:** 10.3389/fped.2018.00038

**Published:** 2018-02-27

**Authors:** Abigail Beane, Priyantha Lakmini Athapattu, Arjen M. Dondorp, Rashan Haniffa

**Affiliations:** ^1^Network for Improving Critical Care Systems and Training (NICST), Colombo, Sri Lanka; ^2^Ministry of Health, Nutrition and Indigenous Medicine, Colombo, Sri Lanka; ^3^Mahidol Oxford Tropical Medicine Research Unit, Faculty of Tropical Medicine, Mahidol University, Bangkok, Thailand; ^4^Centre for Tropical Medicine, Medical Sciences Division, University of Oxford, Oxford, United Kingdom

**Keywords:** low- and middle-income countries, low resource settings, researchers, pediatric critical care, support of research, surveys and questionnaires, intensive care unit

Von Saint Andre-Von Arnim and colleagues noted that LMIC clinicians should be empowered to influence local and global research agendas for critically unwell children (1). We too can report that clinicians trained in LMIC acknowledge the need for systematic gathering of outcome data in improving services and endorse the role that non-LMIC collaborators can play in contributing to training, surveillance, and research (2). Interestingly, these perceptions were more strongly held when compared to High Income Country (HIC) counterparts with experience in LMIC settings. These findings perhaps point to untapped opportunities to upskill LMIC clinicians to build equitable research and training partnerships with their non-LMIC counterparts.

Network for Improving Critical Care Systems and Training (NICST) is an LMIC-based organization working collaboratively, since 2012, with clinical teams to build capacity for research, training, and continuous audit to improve patient outcomes (3). A collaboration between clinicians, researchers, and educational experts based in HICs and LMIC, the network, funded in part by a UK grassroots charity of the same name, links over 110 state and private sector hospitals and has trained over 4,500 nurses and doctors in acute and critical care skills.

The NICST platform, a clinician-led mobile electronic health information initiative, is an example of a setting-adapted national registry for critically unwell adults, children, and neonates in Sri Lanka and beyond. Output from the registry supports a critical care bed availability system that facilitates access to and utilization of resources and provides information on post-hospital outcomes (4). Mobile applications linked to the platform improve the availability of information essential for the care of individual patients and enable practical training for nurses and doctors (5). Partnered with institutions based in HIC (UK and the Netherlands), it is creating educational opportunities (MSc, PhD pathways) (6, 7) and undertaking frontline quality improvement projects in Sub-Saharan Africa and South Asia.

Creating sustainable partnerships that harness the power of the existing LMIC-based network enables equitable exchange of expertise and fosters greater understanding of setting-specific research priorities. We anticipate that these successful collaborations coupled with rising awareness of the importance of high-quality surveillance systems in LMIC will somewhat help address the challenges currently experienced by LMIC-based researchers approaching traditional funding streams. We believe our model provides a template for promoting setting-relevant research, which can enable successful south-to-south (and perhaps south-to-north!) collaborations.

## Author Contributions

RH and AB wrote the first draft of the manuscript. AD and PA approved and improved the manuscript. All the authors approved the contents.

## Conflict of Interest Statement

The authors declare that the research was conducted in the absence of any commercial or financial relationships that could be construed as a potential conflict of interest.
